# Multi-Omics Characterization of Peripheral Blood Molecular Profiles in Hypertensive Aging *Ailuropoda melanoleuca* with Levamlodipine Intervention: Exploratory Analysis of *ACE2* and Time-Resolved Transcriptomic Patterns

**DOI:** 10.3390/ani16142116

**Published:** 2026-07-08

**Authors:** Yan Zhu, Qian Tao, Chengyao Li, Shanshan Ling, Ming Wei, Mengfang Yang, Danyu Chen, Desheng Li, Caiwu Li, Chengdong Wang

**Affiliations:** State Forestry and Grassland Administration Key Laboratory of Giant Pandas, China Conservation and Research Center for the Giant Panda, Chengdu 610000, China; zhuyan_494062079@163.com (Y.Z.);

**Keywords:** captive giant pandas, hypertension, RNA-seq, ATAC-seq, scRNA-seq

## Abstract

Hypertension threatens the health of aging captive giant pandas, yet its molecular signatures remain poorly characterized. Here, multi-omics sequencing was applied to explore the molecular regulatory process of hypertension and the pharmacodynamic effect of levamlodipine on this endangered species. Six aged giant pandas were divided into hypertensive and normotensive groups (three hypertensive and three normotensive pandas) based on clinical phenotypes and blood pressure measurements. A multi-omics approach was employed, including blood RNA-seq, Assay for Transposase-Accessible Chromatin with high-throughput sequencing (ATAC-seq), single-cell RNA-seq (scRNA-seq) of peripheral blood mononuclear cells, and time-series RNA-seq following levamlodipine administration. Our transcriptomic analysis revealed a statistically significant decline in ACE2 transcript abundance (Padj < 0.05), which suggests a possible shift in renin–angiotensin system signaling linked to hypertensive status in giant pandas. Single-cell analysis of 88,693 cells revealed that hypertension-associated genes were predominantly enriched in monocytes and T cells, implicating immune cell activation. Time-dependent transcriptional changes after levamlodipine administration. Temporal gene dynamics showed early activation of metabolic pathways followed by delayed inhibition of ion channels and calcium signaling. This study provides a transcriptional molecular perspective into the pathogenesis of hypertension in giant pandas, which is conducive to developing more effective antihypertensive treatment strategies, thereby protecting the health of this endangered species.

## Simple Summary

This study addresses high blood pressure in six aging captive giant pandas (*Ailuropoda melanoleuca*), a condition that threatens their health. The goal was to uncover the molecular signatures and test how a blood pressure medication works in this endangered species. Using three sequencing methods—Assay for Transposase-Accessible Chromatin with high-throughput sequencing to map gene regulatory regions, single-cell RNA-seq to analyze individual immune cells, and time-series RNA-seq to track gene changes over time—the study found that high blood pressure is linked to reduced activity of a specific gene and activation of certain immune cells. Time-dependent transcriptional changes occur after levamlodipine administration. These findings provide crucial knowledge for developing better treatments, helping protect the health of giant pandas.

## 1. Introduction

Hypertension is a widespread chronic disease worldwide, mainly characterized by persistently high arterial blood pressure. It not only has a high incidence in humans but is also a major risk factor for heart disease [1]. Hypertension can be divided into primary and secondary types, with most cases being primary hypertension of unknown origin. Factors such as genetic predisposition, dietary habits, high salt intake, obesity, and lack of exercise are considered significant triggers of hypertension [2]. In recent years, hypertension in animals, particularly wild ones, has increasingly garnered attention. Although there have been few systematic studies, reports have indicated that many animals in captivity experience health issues similar to those in humans, such as hypertension [3]. During the conservation of endangered animals, health monitoring shows that hypertension may be linked to shifts in their living conditions and diet. For instance, the food provided to captive animals by caretakers might lack the variety of natural foods available in the wild, potentially leading to hypertension, which underscores the importance of addressing these dietary adjustments in captivity. In addition, animals subjected to mental stress or environmental changes, such as long-distance transportation and habitat alterations, are prone to hypertension-like symptoms [4]. In recent years, multiple cases of giant pandas have shown signs of elevated blood pressure, especially elderly giant pandas, severely affecting their health. For example, studies have shown that chronic kidney disease in captive elderly giant pandas can lead to persistent hypertension, and this hypertension can cause myocardial damage and heart failure [5]. Furthermore, studies have shown that the stress-inducing operation in captivity can cause acute hypertension in giant pandas. This confirms that giant pandas have a predisposition to hypertension and that the stress from the captivity environment leads to abnormal blood pressure [6]. All along, we have been continuously measuring the blood pressure of elderly giant pandas and providing antihypertensive treatment for hypertensive giant pandas. However, research on the molecular signatures responsible for hypertension in giant pandas has not yet been reported. 

ATAC-seq and RNA-seq are two powerful high-throughput sequencing technologies. Combining them can comprehensively reveal the transcriptional regulatory mechanisms and gene expression dynamics of cells. ATAC-seq identifies accessible regions by detecting open chromatin areas, thus inferring the locations of potential regulatory elements such as promoters and enhancers, etc [7]. By analyzing chromatin accessibility, researchers can identify which genes might be activated or suppressed. RNA-seq directly measures mRNA transcription levels within cells, offering a comprehensive view of gene expression [8]. Combining ATAC-seq and RNA-seq results allows for the analysis of gene regulatory networks from different dimensions: ATAC-seq provides information on the accessibility of regulatory regions, while RNA-seq offers actual data on gene expression levels. Together, they allow for a more in-depth analysis of which chromatin accessibility changes are potentially related to changes in gene expression.

scRNA-seq is a breakthrough technology that allows for the detailed analysis and comprehension of the gene expression profiles at the cellular level. It enables an in-depth exploration of cellular interactions, identification of rare cell types, and monitoring changes in cellular states. It is a crucial instrument for revealing the mechanisms of tissue development, immune responses, and diseases, including cancer [9,10].

scRNA-seq technology has been demonstrating great potential in hypertension research, revolutionizing the field by illuminating complex pathological mechanisms with the precision of single-cell analysis [11]. Hypertension is a complex disease influenced by multiple factors, involving the intricate interplay between various tissues and cell types during its development [12]. Bulk cell research methods provide an overview of overall expression, yet cannot reveal cell-to-cell heterogeneity and molecular features specific to individual cells [13]. scRNA-seq technology addresses the limitation of traditional methods in analyzing gene expression in bulk samples. By performing single-cell analysis of gene expression, scRNA-seq not only compensates for these limitations but also provides novel insights into advancing hypertension research [14]. In recent years, researchers have used scRNA-seq to discover various cell subpopulations involved in blood pressure regulation and their specific expression patterns in the cardiovascular system [12]. Through analyses of transcriptome data from singular heart and artery cells, the dynamic expression changes in endothelial cells and smooth muscle cells during the onset of hypertension have been identified [11]. In particular, the role of endothelial cells in the remodeling and dysfunction of blood vessels is regarded as one of the key factors in the occurrence of hypertension [15]. The pathological process of hypertension is significantly influenced by inflammatory responses, and scRNA-seq technology reveals the complex roles of immune cells such as macrophages and T cells in hypertension [12]. For example, these cells modulate vasoconstriction within inflammatory environments, thereby affecting overall blood pressure control [15]. These studies not only deepen our understanding of the molecular mechanisms of hypertension but also provide a foundation for developing new therapeutic targets and diagnostic markers. However, as of now, there have been no reports on using scRNA-seq to study the molecular mechanisms of hypertension onset in giant pandas. 

This study employed multi-omics sequencing to investigate the molecular mechanisms underlying hypertension and the pharmacodynamic effects of levamlodipine in aged giant pandas. Six elderly individuals were classified into hypertensive and normotensive groups (*n* = 3 each) based on clinical phenotypes and blood pressure readings. Integrated multi-omics analyses were performed, including bulk RNA-seq, ATAC-seq, single-cell RNA-seq (scRNA-seq) of peripheral blood mononuclear cells, and time-series transcriptomic profiling during levamlodipine treatment. The results demonstrated significantly decreased expression of *ACE2* (Padj < 0.01), suggesting dysregulation of the renin–angiotensin system. The single-cell analysis of 88,693 cells further revealed that hypertension-related genes were mainly enriched in monocytes and T cells, implying the involvement of immune cell activation. Moreover, levamlodipine induced time-dependent transcriptional alterations, characterized by early activation of metabolic pathways followed by late suppression of ion channels and calcium signaling. This is the first multi-omics analysis of hypertension in giant pandas, the first PBMC scRNA-seq dataset in hypertensive giant pandas and the first transcriptomic evaluation after levamlodipine treatment in this species. These findings not only clarify some of the molecular regulatory process of hypertension in giant pandas, but also offer new insights into clinical treatment strategies, thereby providing crucial scientific evidence for safeguarding the health of this endangered species.

## 2. Materials and Methods

### 2.1. Animals

All giant pandas included in this study were housed at the Dujiangyan Base of the China Conservation and Research Center for the Giant Panda. To minimize the influence of age and gender variables, the pandas we selected were all elderly ones (officially defined as pandas over 20 years old as being elderly), and both male and female individuals were included. The pandas were in good condition during the blood collection process. We treated hypertensive giant pandas with levamlodipine besylate tablets (Shihuida, Chengdu, China, H19991083) and conducted time-series RNA-seq. Whole blood samples from 6 giant pandas (Table 1) were collected using EDTA anticoagulant collection tubes (Meidike, Shenzhen, China) and PAX gene^®^ blood RNA tubes (BD Biosciences, Milpitas, CA, USA), respectively, and mixed upside down. Fresh whole blood samples were temporarily stored at 4 °C and transported to the laboratory within 2 h after collection, and PBMC isolation (TBD Science, Tianjin, China) was conducted immediately upon arrival using pre-cooled lymphocyte separation medium via density gradient centrifugation. After isolation, viable PBMCs were cryopreserved with standard freezing medium via slow cooling and temporarily stored at −80 °C before long-term liquid nitrogen storage to retain cell integrity. For samples collected using PAXgene^®^ Blood RNA Tubes (BD Biosciences, Milpitas, CA, USA), the tubes were first kept at 18–25 °C for 2 h, then stored overnight at −20 °C, and finally placed in a −80 °C freezer for preservation. RNA-seq, ATAC-seq and scRNA-seq were performed on six giant panda samples.

### 2.2. Blood Pressure Measurement and Hypertension Criteria

The systolic and diastolic blood pressures of each giant panda were measured daily at a fixed time (15:00) using a blood pressure monitor (Omron, Japan, HEM-7211). A total of 3 daily technical measurements per panda were averaged to get a daily mean, then 3 daily means across three days were further averaged to produce one single representative blood pressure value for each giant panda. Only these six individual aggregated means (3 hypertensive, 3 normotensive) were used to calculate intergroup *p*-values. All animals maintained a natural sitting posture throughout the whole measurement process, with no forced restraint or sedation applied. A small-size adjustable cuff matching giant panda forearm circumference was firmly and evenly wrapped around the midpoint of the right forearm of each subject. All operations were completed by professional, specially trained full-time keepers with long-term daily care experience for these giant pandas; the pandas are fully familiar with routine blood pressure detection as a regular health management item and cooperate voluntarily under calm, low-stress conditions. This human-origin Omron HEM-7211 monitor has been deployed for daily giant panda blood pressure surveillance at our base for many years, and long-term historical monitoring data demonstrate stable, repeatable pressure readouts when operated with standardized forearm cuff placement in captive giant pandas. The average systolic and diastolic blood pressure values calculated from nine repeated measurements per individual were used to assign each giant panda to the hypertensive or normotensive cohort.

At present, no globally unified validated diagnostic standard for hypertension has been established for giant pandas. Previous preliminary research reported the baseline physiological blood pressure range of healthy captive giant pandas as systolic blood pressure 147.53 ± 28.04 mmHg and diastolic blood pressure 100.48 ± 24.19 mmHg, which was used as the reference baseline for this study. Combined with decades of continuous routine health monitoring and clinical observation of giant pandas at our breeding station, we summarized an empirical clinical threshold for screening individuals with hypertensive manifestations: giant pandas with persistent resting systolic blood pressure > 160 mmHg and diastolic blood pressure > 130 mmHg frequently develop typical hypertension-related clinical signs.

### 2.3. Isolate PBMCs from Giant Pandas

PBMCs were isolated using the Giant Panda PBMC Isolation Kit (LDS1113) produced by Tianjin Haoyang Biological Products Technology Co., Ltd. (Tianjin, China). The specific operation was as follows: Add 5 mL of the separation solution to a 15 mL sterile centrifuge tube, then slowly add 5 mL of the blood sample. Centrifuge at 600× *g* for 25 min at 20 °C. Carefully transfer the plasma layer to the new centrifuge tube A. Carefully transfer the circular white single-nucleus cell layer in the centrifuge tube to the new centrifuge tube B. Add 10 mL of cleaning solution to centrifuge tube B, mix the cells. Centrifuge at 250× *g* for 10 min, discard the supernatant. Use a pipette to aspirate 5 mL of cleaning solution to resuspend the obtained cells. Centrifuge at 250× *g* for 10 min, discard the supernatant. Repeat this cleaning process twice, discard the supernatant, and finally resuspend the obtained cells with 0.5 mL of cleaning solution.

### 2.4. RNA Sequencing and Analysis

Extract total RNA from giant panda peripheral blood samples. RNA integrity was evaluated using an Agilent 2100 Bioanalyzer system; only RNA samples with an RNA integrity number (RIN) ≥ 7 were retained for library construction. A total of 1 μg intact total RNA was used as the starting input for each library, and strand-specific RNA sequencing libraries were constructed using the Illumina^®^ RNA Library Prep Kit (New England Biolabs, Ipswich, MA, USA). In simple terms, Use Oligo (dT) magnetic beads to enrich mRNA with poly-A tails, and then randomly break the mRNA in Fragment Buffer using divalent cations. End repair treatment was carried out on the purified double-stranded complementary DNA (cDNA), followed by A-tailing and ligation with sequencing adapters. Fragments ranging from 370 to 420 bp in length were separated and enriched using AMPure XP magnetic beads, which were then utilized once more to purify the amplified products from PCR reactions, ultimately yielding the sequencing library. RNA-seq libraries were sequenced on the Illumina NovaSeq 6000 sequencing platform (Novagene, Beijing, China), with a unified paired-end 150 bp (PE150) sequencing strategy. Each sample was sequenced to approximately 8 Gb clean data, and all RNA-seq datasets used the same strategy. Subsequent to quality filtering of raw sequencing reads, HISAT2 software (v.2.2.1) was adopted to align the processed clean sequencing reads against the reference genome sequences. The reference genome and gene annotation of *Ailuropoda melanoleuca* were retrieved from Ensembl release 110; the genome assembly version was ASM200744v2 (NCBI assembly accession GCA_002007445.2). Primary-assembly FASTA genome sequences were downloaded from https://ftp.ensembl.org/pub/release-110/fasta/ailuropoda_melanoleuca/dna/ (accessed on 1 April 2026), and the matched official gene annotation GTF file (Ailuropoda_melanoleuca.ASM200744v2.110.gtf.gz) was obtained at https://ftp.ensembl.org/pub/release-110/gtf/ailuropoda_melanoleuca/ (accessed on 1 April 2026). The sequencing quality indicators are shown in Table S1. The abundance of messenger RNA transcripts was quantified using the FPKM algorithm (Fragments Per Kilobase of exon model per Million mapped fragments). Given that all samples were set up with three biological replicates, the R software (v.4.3.3) package DESeq2 (v.1.42.0) was applied to conduct differential expression analysis for each comparison group based on raw counts [16]. The Benjamini & Hochberg algorithm was adopted to adjust all *p*-values, and the adjusted *p*-value < 0.05 and |log2FoldChange| ≥ 2 were defined as the standard threshold to screen genes with obvious expression differences, which followed the default parameters described in reference [17]. The clusterProfiler program was utilized to implement Gene Ontology (GO) and Kyoto Encyclopedia of Genes and Genomes (KEGG) enrichment analyses targeting differentially expressed genes (DEGs), as documented in the literature [18]. Note that when conducting the differential expression analysis of time-series RNA-seq, all samples were repeatedly collected from the same three hypertensive giant pandas across multiple post-drug time points, forming a classic repeated-measures longitudinal dataset. To account for inherent transcriptional differences between individual animals and remove within-animal correlation bias caused by repeated sampling, we incorporated individual animal ID as a blocking covariate in the statistical model. The final DESeq2 design formula was specified as: design = ~ animal + timepoint. All pairwise differential expression comparisons between different time points were performed under this full adjusted model, rather than simple two-group designs that ignore the repeated sampling structure.

### 2.5. ATAC Sequencing and Analysis

After adding Tn5 transposase to the nuclei suspension for the transposition reaction (37 °C, 30 min), equimolar concentrations of Adapter1 and Adapter2 are added after the reaction is completed for PCR amplification of the library using the ATAC-seq_Novogene Kit. Following PCR amplification, AMPure magnetic beads were used to purify the constructed library, and the Qubit detection system was adopted to assess library quality. Library construction and transcriptome sequencing services were completed by Tianjin Novogene Bioinformatics Technology Co., Ltd. (Beijing, China) on the Illumina NovaSeq sequencing platform, with 150 bp paired-end sequence fragments generated during the sequencing process. Raw sequencing data in FASTQ format (raw reads) were preprocessed using fastp software (v0.20.0). Sequencing adapters were eliminated in this processing procedure to acquire clean reads, reads with more than 6 bases as N, and other low-quality reads (reads where bases with a quality score less than 15 exceed 40% of the base count for that read, and reads shorter than 18 bp after trimming) from the raw data. BWA (v.0.7.12) software was used to align clean reads with the reference genome, removing reads from mitochondrial DNA. Screening was performed on sequencing reads to retain high-quality sequences with MAPQ ≥ 13. Reads with abnormal pairing information and copies amplified through PCR were excluded in this filtering procedure. Further analysis was conducted using reads with unique mapping (MAPQ ≥ 13) and those with duplicate data removal. MACS2 software (version 2.2.7.1) was utilized to identify all binding peaks with the following complete formatted command: macs2 callpeak -q 0.05 --call-summits --nomodel --shift -100 --extsize 200 --keep-dup all. Peaks with q-value < 0.05 were retained according to the threshold set in the MACS2 command. ChIPseeker (v.1.48.0) software was used to obtain peak-related genes and using ChIPseeker to annotate the genomic regions of the peaks [19]. For differential chromatin accessibility analysis across hypertensive and normotensive groups, peaks identified from all individual samples were merged to generate a comprehensive consensus peak catalog, containing both group-specific peaks and shared constitutive peaks detected across hypertensive and normotensive giant pandas. The full consensus peak set was used for downstream quantitative differential analysis using DiffBind (v3.8.4). This package implements negative binomial generalized linear models to estimate variance across biological replicates and perform multiple-testing correction via the Benjamini–Hochberg method. RPM (reads per million) values were calculated solely for sample clustering and visualization and were not adopted as a statistical threshold to define differentially accessible peaks (DAPs). DAPs were identified using a dual filtering standard: adjusted FDR < 0.05 and |log2 fold change| > 1. ChIPseeker was further used to annotate target genes linked to DAPs, with a focus on peaks localized to gene promoter regions. Functional enrichment of DAP-associated genes was then conducted for Gene Ontology (GO) terms.

### 2.6. Single-Cell RNA Sequencing and Analysis

The cell suspension was loaded into the Chromium microfluidic chip designed for 3’ capture, followed by cell barcoding operation on the 10 × Chromium Controller device manufactured through 10× Genomics. Next, reverse transcription was conducted on RNA molecules extracted from individually barcoded cells. In accordance with the official operating guidelines, the Chromium Single Cell 3’ v2 reagent kit (10× Genomics) was adopted to complete sequencing library construction. Subsequent sequencing procedures were carried out on Illumina sequencing instruments following the manufacturer’s standard protocols. FASTQ files containing raw sequencing reads output from the Illumina platform were preprocessed with Trimmomatic software (v.0.36) under customized filtering parameters. A sliding window spanning four nucleotides was applied to scan each read; sequences were cut off if the average base quality value within the window fell below 10 (parameter setting: SLIDINGWINDOW: 4: 10). All terminal bases with low quality scores or ambiguous N bases (quality threshold < 3) were also trimmed via the TRAILING:3 function. Two alternative strategies were available for the elimination of adapter contamination sequences: 1. align with adapter sequences, with more than 7 bases matched and mismatches = 2; 2. remove non-overlapping parts when the base score of the overlap between read1 and read2 is greater than 30 (ILLUMINACLIP: adapter. fa: 2: 30: 7). Discard reads with length shorter than 26 bases and those that fail the pairing step. Remaining reads that pass all filtering stages are referred to as “filtered reads,” forming the basis for all subsequent analyses. Finally, basic quality control and filtering of clean reads were performed using fastp. Demultiplexing, genome alignment, feature counting, and matrix generation for single-cell RNA-seq data were performed using 10× Genomics Cell Ranger (v.7.1.0) (https://support.10xgenomics.com/single-cell-gene-expression/software/pipelines/latest/what-is-cell-ranger) (accessed on 19 April 2026). A custom single-cell reference index was built based on the giant panda reference genome assembly ASM200744v2 (GCA_002007445.2) and Ensembl release 110 gene annotation. Standard default parameters were applied for Cell Ranger count and subsequent Cell Ranger reanalyze workflows, including dimensionality reduction, clustering, and gene expression quantification. All downstream single-cell analyses were performed using Cell Ranger and Seurat software. Differential expression analysis to identify cell-type-specific marker genes was performed using the edgeR package (v.4.0.16) [20]. The adjusted *p*-value < 0.05 and |log2FoldChange| ≥ 0.5 were defined as the standard threshold for screening genes with obvious expression differences. Before statistical testing, raw UMI counts from the Seurat-filtered single-cell matrix were aggregated into pseudo-bulk profiles by individual giant panda and cell subtype; raw counts of all cells originating from the same panda and cell population were summed. Each giant panda was regarded as one independent biological replicate, and the aggregated pseudo-bulk count matrix was used as the input for edgeR to avoid pseudoreplication caused by individual cells. Functional enrichment analyses of Gene Ontology (GO) and KEGG pathways were subsequently carried out with the clusterProfiler (v.3.14.0) R package (v.4.3.3).

### 2.7. Statistical Analysis

Excel 2019, GraphPad Prism 7.0 and Adobe Illustrator 2020 software were used for the analysis and plotting of blood pressure measurement data. Data were shown as the mean ± S.D., and values of *p* < 0.05 were considered statistically significant (* *p* < 0.05, ** *p* < 0.01).

## 3. Results

### 3.1. Hypertension in Captive Giant Pandas

**Figure 1:**
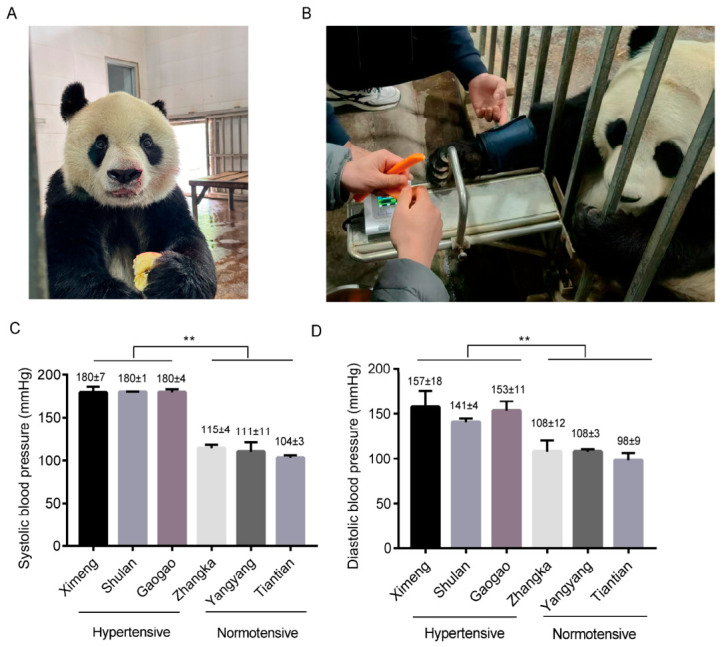
Blood pressure measurement of giant pandas. (**A**) A photo of an elderly giant panda (Gaogao) with severe nosebleed; (**B**) measurement of the blood pressure of a giant panda (photos by Rui Cheng); (**C**) statistical bar chart of systolic blood pressure (mmHg) in six individual giant pandas; three individuals (Ximeng, Shulan, Gaogao) were classified as hypertensive, while the other three (Zhangka, Yangyang, Tiantian) were normotensive; (**D**) statistical bar chart of diastolic blood pressure (mmHg) across the same six giant panda individuals. These data are expressed as mean ± S.D. ** *p* < 0.01 compared with control.

In recent years, as the age of giant pandas increases, some elderly captive giant pandas have experienced nosebleeds (Figure 1A). Through multiple blood pressure measurements (Figure 1B), the results showed that the systolic blood pressure of some pandas reached as high as 186 ± 2 mmHg, and the diastolic blood pressure reached as high as 147 ± 3 mmHg, while the systolic blood pressure of some pandas was only 117 ± 3 mmHg, and the diastolic blood pressure was 107 ± 2 mmHg (Figure 1C,D). Therefore, we divided these pandas into the hypertensive group (Shulan, Gaogao, Ximeng) and the normotensive group (Zhangka, Tiantian, Yangyang).

### 3.2. Analysis of RNA-seq Data from the Blood of Hypertensive Giant Pandas

**Figure 2:**
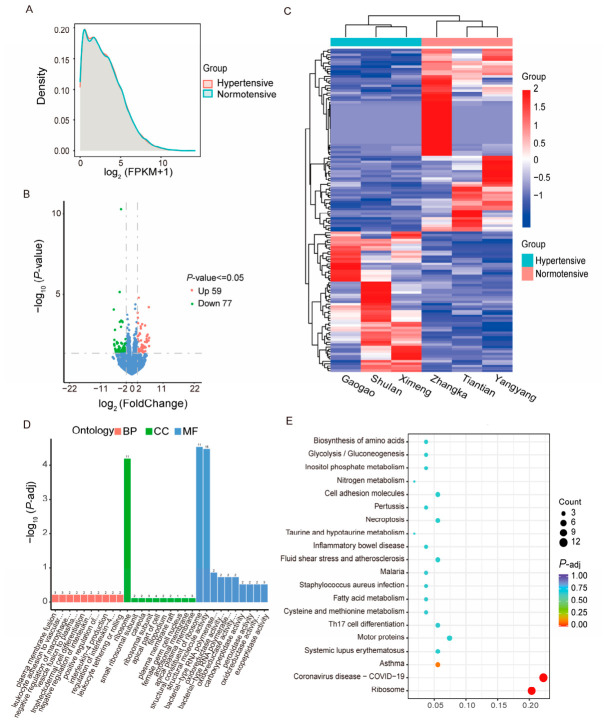
Functional characterization and differential expression profiling of DEGs. (**A**) Density distribution curve reflecting gene FPKM expression levels; (**B**) statistical summary of DEGs identified in giant pandas by comparing the hypertensive cohort against the normotensive cohort, with screening criteria set as adjusted *p*-value < 0.05 and|log2FoldChange| ≥ 2; (**C**) hierarchical clustering heatmap plotting all DEGs derived from the hypertensive and normotensive panda groups, where the red-to-blue color gradient corresponds to normalized FPKM expression values; (**D**,**E**) GO and KEGG enrichment assessments for DEGs filtered by *p*-value < 0.05.

RNA sequencing technology was adopted to characterize the mRNA transcription profiles in blood samples collected from giant pandas suffering from hypertension. The distribution and density curves of FPKM values illustrated that the overall mRNA transcriptional abundance exhibited high consistency across all tested giant panda individuals (Figure 2A). When contrasted with the normotensive cohort, 136 differentially expressed genes (DEGs) were screened out in the hypertensive group; among these genes, 59 transcripts displayed elevated expression, while the remaining 77 genes were suppressed (Figure 2B). Hierarchical clustering analysis further visualized the distinct expression trends of these DEGs, and the three biological duplicate samples within each treatment group clustered tightly together (Figure 2C). Subsequently, GO and KEGG enrichment analyses were carried out independently. In general, differentially expressed genes (DEGs) exhibited prominent enrichment results under the threshold of adjusted *p*-value < 0.05—for the cellular component category, the enriched term was ribosome—while in the molecular function category, the significantly enriched entries included ribosome structural component and structural molecular activity (Figure 2D). We performed KEGG functional enrichment of DEGs and observed prominent enrichment in ribosome-associated pathways, alongside several database entries annotated to viral infection processes (Figure 2E). Given that our study focuses on hypertension in captive giant pandas without relevant viral infection intervention or clinical phenotypes, we did not conduct targeted biological interpretation of these viral/disease-related KEGG terms.

### 3.3. High-Quality Sequencing Data from ATAC-seq

**Figure 3:**
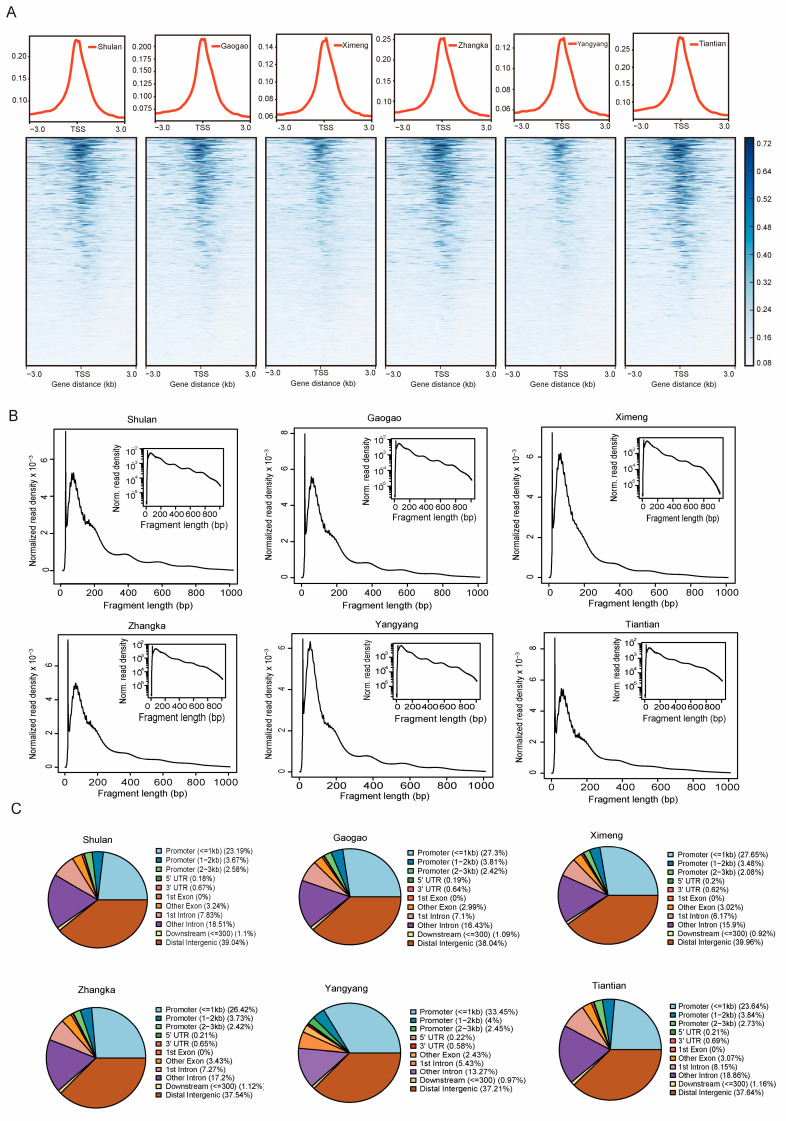
Quality analysis of ATAC-seq data. (**A**) Density distribution graphs and heatmaps illustrating aligned sequencing reads within the 3-kilobase genomic segments surrounding TSS; (**B**) curves showing the length distribution of inserted DNA fragments derived from ATAC-seq specimens; (**C**) distribution map of peaks for each sample in gene functional elements.

We collected fresh blood samples from hypertensive group and normal blood pressure group respectively, and performed ATAC-seq sequencing. The ATAC-seq analysis of each sample generated clean reads ranging from 14.07 and 21.98 Gb, with an average size of 18.33 Gb. Comparing with the reference genome, it was shown that read mapping rates were above 91%, indicating a high sequencing quality (Table S2), and other key quality metrics, such as the FRiP score, were all listed in Table S2. Furthermore, the sequencing reads obtained from ATAC-seq displayed obvious enrichment around transcription start sites (TSSs) (Figure 3A), which verified the good quality of the sequencing data. As for the fragment length distribution of ATAC-seq specimens, the majority of inserted DNA fragments presented short lengths, corresponding to nucleosome-free open chromatin domains (Figure 3B). A Spearman correlation heatmap of peak read counts proves strong reproducibility between biological replicates, with all correlation coefficients above 0.86 (Figure S1). Afterwards, functional annotation was conducted on all identified open chromatin regions. The annotation outcomes revealed that the bulk of chromatin peaks fell within promoter and intergenic zones, whereas intronic regions harbored only a minor fraction of peaks (Figure 3C). This distribution feature suggests that transcription factors preferentially interact with promoter sequences adjacent to TSSs. The above results all indicate high-quality ATAC-seq data.

### 3.4. Differential Chromatin Accessibility Analysis of ATAC-seq

**Figure 4:**
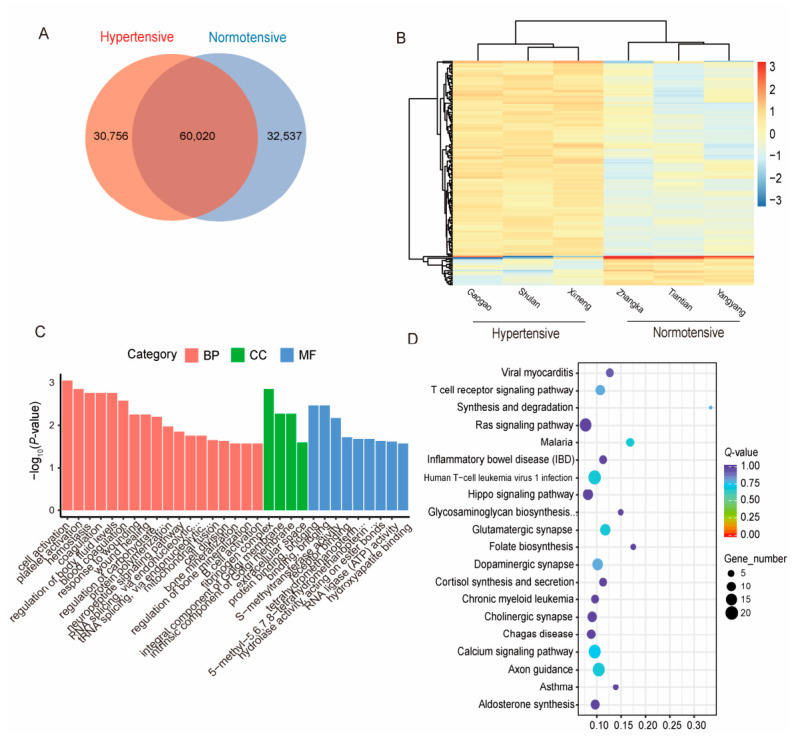
Differential analysis of ATAC-seq data. (**A**) Venn diagram showing the peak difference between hypertensive group and normotensive group, and the numbers in the pie chart represent the number of peaks; (**B**) hierarchical clustering diagram of the RPM values of peaks in the hypertensive group and normotensive group; (**C**,**D**) GO and KEGG enrichment analysis of genes related to different peaks with corrected *p*-value < 0.05.

Next, differential chromatin accessibility analysis was performed on ATAC-seq peak signals. Consensus peaks merged across all samples yielded a total of 60,020 shared peaks present in both hypertensive and normotensive groups; 30,756 peaks were uniquely detected in the hypertensive group, and 32,537 peaks were exclusive to the normotensive group (Figure 4A). All merged consensus peaks covering both group-unique and shared constitutive chromatin regions were included in quantitative differential accessibility testing via DiffBind. Hierarchical clustering based on normalized peak accessibility signals clearly separated the two experimental cohorts, with all three biological replicates from each group tightly clustered within their respective treatment group, confirming high reproducibility of chromatin accessibility profiles (Figure 4B). Independent Gene Ontology (GO) and KEGG pathway enrichment analyses were performed on genes linked to differential ATAC-seq peaks. It should be noted that the assignment of distal intergenic accessible chromatin peaks to putative target genes bears inherent uncertainty, and all enrichment results presented here should be interpreted cautiously as preliminary correlative observations. Generally speaking, with corrected *p*-value < 0.05, genes related to different peaks significantly enriched to cell activation, platelet activation in biological process, fibrinogen complex in cellular components, and protein binding, bridging in molecular function (Figure 4C). The KEGG analysis showed the relevant signaling pathways were mainly concentrated in axon guidance and glutamatergic synapse (Figure 4D).

### 3.5. Integration Analysis of ATAC-seq and RNA-seq

**Figure 5:**
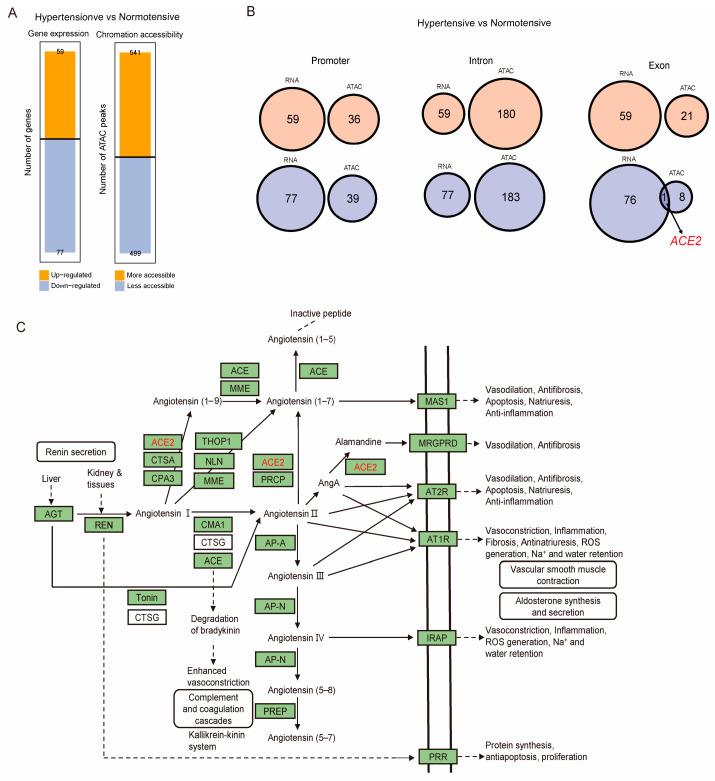
Comprehensive integrative analysis of ATAC-seq and RNA-seq data. (**A**) Statistics of differentially expressed genes (DEGs) from RNA-seq and differentially accessible peaks (DAPs) from ATAC-seq; (**B**) Venn diagrams showing the overlap of genes associated with DEGs and DAPs distributed in different genomic regions (promoter, exon, and intron). Orange circles represent upregulated genes/associated genes, and blue circles represent downregulated genes/associated genes; (**C**) Enriched KEGG metabolic pathway analysis of integrated DEGs and DAP-related genes.

To uncover potential molecular signatures related to hypertension, we performed an integrative multi-omics analysis combining RNA-seq-derived differentially expressed genes (DEGs) and ATAC-seq-derived differentially accessible peaks (DAPs). The left panel of Figure 5A summarizes transcriptional variation between groups: 59 genes were significantly upregulated (yellow) and 77 genes were downregulated (blue) in the hypertensive giant pandas relative to normotensive controls. The right panel illustrates chromatin accessibility dynamics, with 541 peaks gaining accessibility (yellow) and 499 peaks losing accessibility (blue) under hypertensive conditions. We further overlapped DEGs and DAP-associated genes across three genomic compartments (promoter, intron, exon). Only one overlapping gene (*ACE2*) was identified, with its corresponding differential accessible peak overlapping an exon of *ACE2*. (Figure 5B). Notably, *ACE2* displayed significant transcriptional downregulation in whole blood RNA-seq data (|log_2_ FoldChange| = 6.38, *p*-value < 0.05), and the differential ATAC-seq peak overlapping an exon of the *ACE2* gene also presented reduced chromatin accessibility in hypertensive giant pandas, meaning consistent downregulation signals were observed at both transcriptional and chromatin accessibility levels. Importantly, this candidate gene signature comes with two key interpretive limitations: no orthogonal qPCR validation was conducted to confirm the differential expression of ACE2, and all sequencing data were generated from peripheral blood leukocytes instead of disease-relevant cardiovascular, renal or vascular tissues that directly mediate blood pressure regulation.

KEGG pathway enrichment further confirmed that ACE2 (angiotensin-converting enzyme 2) acts as a core enzyme governing renin–angiotensin system homeostasis (Figure 5C). ACE2 catalyzes the degradation of angiotensin II and generates vasoprotective angiotensin (1–7), which mediates downstream signaling via the AT2R receptor. Collectively, *ACE2* is indispensable for blood pressure maintenance and cardiovascular protection; it balances vasoactive peptide metabolism and exerts anti-inflammatory and vasodilatory effects via the angiotensin regulatory cascade.

### 3.6. Single-Cell RNA-Sequencing and Clustering Analysis

**Figure 6:**
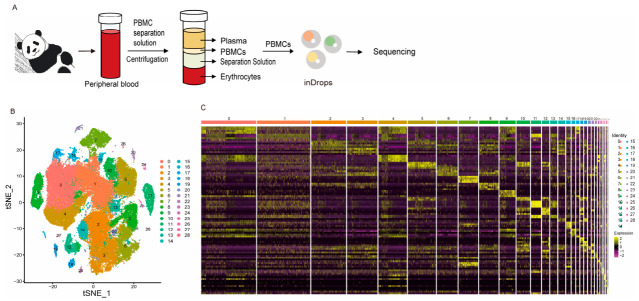
Single-cell RNA-seq data processing and unsupervised clustering of giant panda peripheral blood mononuclear cells (PBMCs). (**A**) Schematic workflow of single-cell RNA-seq analysis for giant panda PBMC samples; (**B**) tSNE dimensional reduction plot showing the unsupervised clustering result of 88,693 single cells, which were partitioned into 29 distinct cell clusters; (**C**) heatmap displaying the top differentially expressed genes (DEGs) that distinguish each cell cluster.

To identify the cell types in the blood of giant pandas, we used the 10× Genomics platform to conduct scRNA-seq analysis on PBMCs from the blood of six giant pandas (Figure 6A). In total, 88,693 cells passed the standard quality control (cell filtering) and were selected for further analyses. A total of 24,571 mean reads were detected per cell and 93.08% reads were mapped to the giant panda genome (Table S3). The sum of detected genes across the six individual samples was 103,457, with per-sample gene counts detailed in Table S3, and a median of 1520 genes identified in each cell. Based on the gene expression patterns, these cells were divided into 29 clusters (Figure 6B), and the differential gene expression patterns of each cluster are shown in Figure 6C.

### 3.7. Cell Type Definition and Difference Analysis of scRNA-seq Data

**Figure 7:**
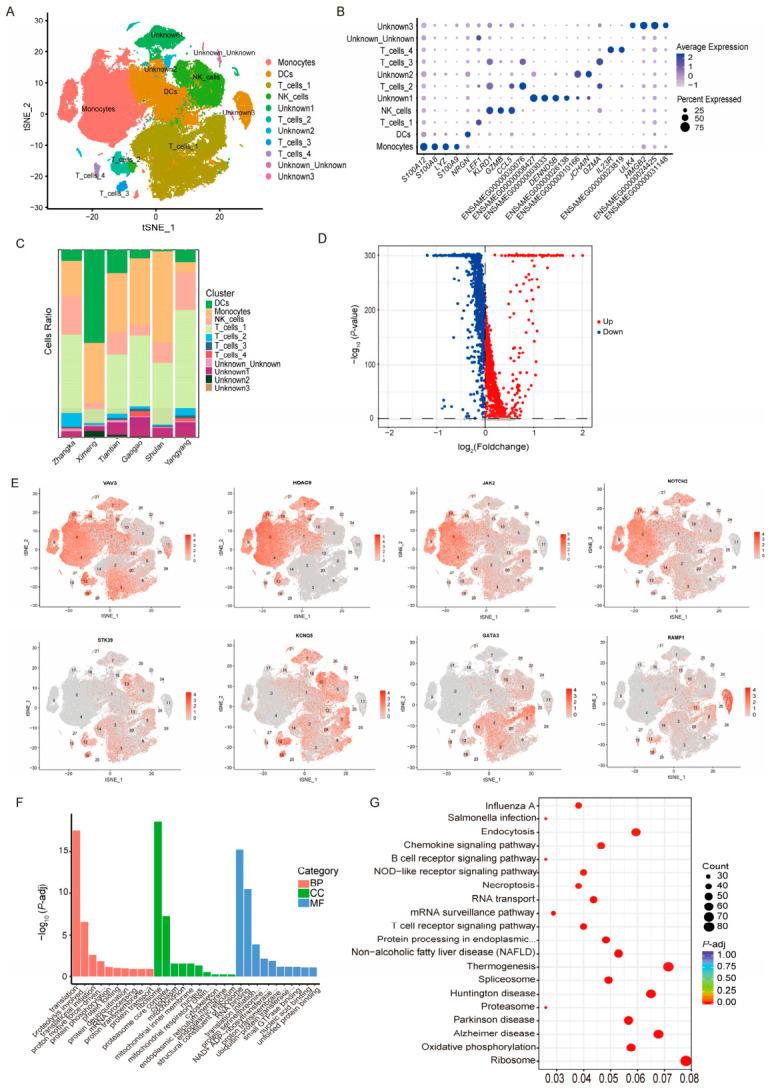
Cell type annotation and comparative transcriptomic analysis based on single-cell RNA-seq data. (**A**) Annotated tSNE plot displaying the assigned cell type labels for each unsupervised cluster; (**B**) dot plot showing the expression patterns of canonical marker genes across all identified cell populations; (**C**) bar chart showing the proportional distribution of each cell type within every sample; (**D**) bar statistics of DEGs screened with the thresholds adjusted *p*-value < 0.05 and |log2FoldChange| ≥ 0.5; (**E**) feature plots presenting the expression signals of hypertension-associated DEGs across individual cell clusters; (**F**,**G**) GO and KEGG functional enrichment analyses for identified DEGs (threshold: adjusted *p*-value < 0.05).

By exploring the known cell-type-specific marker genes, we annotated the cells and classified them into 11 clusters, including monocytes, dendritic cells (DCs), NK cells, various T cell subgroups, and several unknown type cell clusters. Based on the characteristics such as interleukin 7 receptor (IL7R), interleukin 23 receptor (IL23R), granzyme K (GZMK), granzyme A (GZMA), CD7 molecule (CD7), granulin (GRN), T cell receptor delta constant (TRDC), clusters 2, 3, 6, 8, 12, 14, 18–21 are defined as T cells. Based on the characteristics such as S100 calcium binding protein A8 (S100A8), S100 calcium binding protein A9 (S100A9), S100 calcium binding protein A12 (S100A12), clusters 0, 4, 9, 16, 17, 23, 26 are defined as monocytes. Based on top cluster-enriched signature genes, including FRY, neurogranin (NRGN), and calcium dependent secretion activator 2 (CADPS2), clusters 1, 11, 13 and 28 were tentatively annotated as putative dendritic cell (DC) populations. It should be noted that FRY, NRGN and CADPS2 are not well-established canonical mammalian DC markers. Given the absence of a giant panda species-specific immune cell transcriptomic reference atlas, this cell type assignment remains provisional and requires further validation in independent cohorts. Detailed marker comparison and annotation confidence for all clusters are summarized in Table S4. Based on the characteristics such as killer cell lectin like receptor D1 (KLRD1), granzyme B (GZMB), clusters 5, 10 are defined as NK cells and the remaining unknown cells are defined as unknown (Figure 7A,B). 

The proportion distribution results of different clusters in each sample indicated that there were differences in the relative abundance of cell populations among the samples (Figure 7C). The differential expression analysis identified a total of 204 DEGs in the hypertensive group, including 132 upregulated and 72 downregulated genes (Figure 7D). Among these DEGs, multiple genes with reported functions related to blood pressure regulation were screened, including vav guanine nucleotide exchange factor 3 (*VAV3*), histone deacetylase 9 (*HDAC9*), Janus kinase 2 (*JAK2*), notch receptor 2 (*NOTCH2*), serine/threonine kinase 39 (*STK39*), potassium voltage-gated channel subfamily Q member 5 (*KCNQ5*), GATA binding protein 3 (*GATA3*) and receptor activity modifying protein 1 (*RAMP1*). Based on raw single-cell expression signals, we preliminarily observed relatively higher expression signals of these genes in monocytes, T cells and unannotated unknown cell groups (Figure 7E). Finally, GO and KEGG analyses revealed that these DEGs were mainly enriched in pathways such as ribosome and structural constituent of ribosome (Figure 7F,G).

### 3.8. RNA-seq at Various Time Points After Taking Antihypertensive Drugs

**Figure 8:**
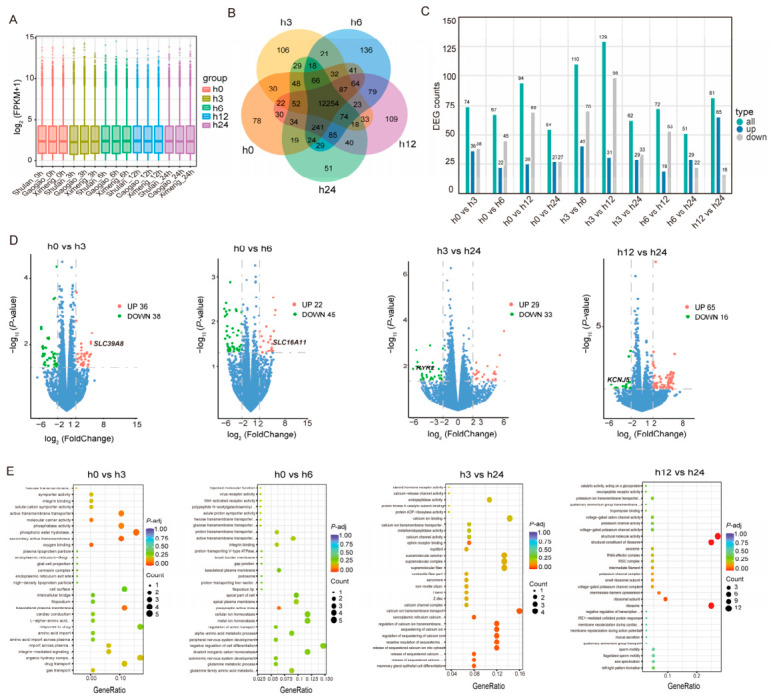
Differential expression profiling and functional enrichment of differentially expressed genes (DEGs) across sequential time points following antihypertensive drug administration. All sampling at each time point was performed on the identical three hypertensive giant pandas for longitudinal comparison. (**A**) Global mRNA FPKM density distribution of peripheral blood transcriptomes sampled at h0, h3, h6, h12 and h24 after levamlodipine administration. Note that temporal transcriptional shifts cannot be definitively assigned to drug effects due to the absence of serial pre-drug self-control baseline samples; (**B**) Venn diagram illustrating unique and overlapping differentially expressed genes detected across sequential sampling time points post levamlodipine treatment. Unique DEG counts only reflect descriptive temporal transcriptional patterns and do not represent quantitative pharmacodynamic signatures of the drug; (**C**) bar graph summarizing the total number of DEGs identified in pairwise time point comparisons; (**D**) volcano plots illustrating DEGs (adjusted *p*-value < 0.05 and |log2FoldChange| ≥ 2) linked to hypertension derived from pairwise comparisons between distinct time points; (**E**) bar plots of GO functional enrichment for DEGs from each pairwise time point comparison.

Next, oral levamlodipine besylate tablets were administered to three hypertensive giant pandas (Shulan, Gaogao, Ximeng). Peripheral blood samples were collected for RNA-seq at a series of sequential time points immediately before drug delivery (h0), and h3, h6, h12, and h24 post-administration. The results of FPKM density showed that mRNA expression levels of each time point were almost consistent (Figure 8A). Venn analysis was performed to quantify differentially expressed genes exclusively detected at each individual time point, yielding 78 unique DEGs at h0, 106 at h3, 136 at h6, 109 at h12, and 51 at h24 (Figure 8B). We further characterized temporal expression trajectories of hypertension-relevant candidate genes across the sequential sampling window after levamlodipine exposure. The transcript abundance of *SLC39A8* showed an upward trend from h0 to h3, while *SLC16A11* expression gradually increased from h0 to h6, corresponding to enrichment of metabolic pathway-related genes at these early-to-middle time points. By contrast, *RYR2* expression exhibited a sustained downward trend from h3 to h24, and *KCNJ5* expression declined continuously from h12 to h24, coinciding with reduced transcriptional activity of genes associated with ion channel and calcium signaling pathways at later sampling stages. (Figure 8C,D). The GO enrichment analysis revealed that in the comparison between h0 and h3, the significantly enriched pathways mainly included oxygen binding and phosphoric ester hydrolase, indicating that at the initial stage, the cells might be engaged in crucial oxygen transportation and phosphate ester hydrolysis activities. In the comparison between h0 and h6, the presynaptic active zone was significantly enriched, suggesting that the cells had already begun to form and regulate the active zone of the synapse. In the comparison between h3 and h24, the significantly enriched pathways mainly included calcium ion transmembrane transport, sequestration of calcium ion, and regulation of calcium ion sequestration, indicating that the storage and regulation of calcium ions within the cell became an important biological event over a longer period of time. In the comparison between h12 and h24, the significantly enriched pathways mainly included ribosome, structural molecule activity, structure component of ribosome, indicating that protein synthesis and ribosome structure activities significantly increased in the later stage (Figure 8E). Overall, the significantly enriched pathways at each time point reflect the unique adaptation processes of cells during different stimulation stages, highlighting the differential regulation of specific molecular mechanisms in response to environmental changes.

## 4. Discussion

Integrated RNA-seq and ATAC-seq data revealed widespread differences in chromatin accessibility and gene transcription between hypertensive and normotensive giant pandas. Notably, the *ACE2* gene exhibited distinct chromatin accessibility alterations within exon regions, implying its critical involvement in hypertension regulation [21]. The renin–angiotensin system (RAS) constitutes an intricate signaling network, where multiple enzymes and receptors coordinate angiotensin metabolism and downstream physiological outcomes. Within this cascade, hepatic angiotensinogen (AGT) is cleaved by renin to generate angiotensin I, which is subsequently processed into bioactive angiotensin II by ACE. Angiotensin II elevates blood pressure via AT1 receptor (AT1R) binding, triggering vasoconstriction, sodium and water retention, and other pro-hypertensive effects. Overall, RAS is central to blood pressure homeostasis and fluid balance [22]. Perturbations to this system, particularly an altered ACE/ACE2 activity ratio, are frequently linked to hypertension and cardiovascular disorders [23]. ACE2 plays a protective role in the cardiovascular system by promoting the production of Ang-(1–7), a metabolite that can promote vasodilation and anti-inflammatory responses through Mas receptors [24]. Therefore, the function of ACE2 is considered to have potential value in treating hypertension and related cardiovascular abnormalities. Current research indicates that abnormal activation of RAS is not only associated with hypertension but also closely related to metabolic syndrome and renal lesions. This is because the AT1 signaling pathway not only affects the contraction of vascular smooth muscle but also regulates the synthesis of aldosterone, a hormone that controls sodium reabsorption and potassium secretion [25]. Further research indicates that a decrease in *ACE2* levels may increase the risk of cardiovascular events, while its moderate expression can slow down the progression of hypertension [26]. Nevertheless, it should be noted that *ACE2* transcript and chromatin accessibility signals detected in peripheral whole blood cannot fully recapitulate local RAS activity in vascular, renal or cardiac tissues. Peripheral blood *ACE2* levels are largely shaped by circulating immune cell proportions and systemic inflammatory status, which represent systemic inflammatory signatures rather than tissue-specific cardiovascular RAS function. Despite this limitation, the coordinated transcriptional and chromatin-level suppression of *ACE2* observed in hypertensive blood samples still reflects systemic RAS disturbance under hypertensive status, providing a molecular signature linked to hypertension progression in giant pandas.

Our multi-omics integration analysis identified coordinated reduced transcription and exon chromatin accessibility of *ACE2* in hypertensive giant pandas, but we acknowledge several limitations of this correlative observation. First, only a single overlapping gene (*ACE2*) was detected within exon regions when intersecting DEGs and DAP-associated genes, which restricts the robustness of our integrated regulatory inference. Furthermore, concurrent changes in mRNA abundance and chromatin accessibility do not constitute definitive proof that chromatin opening status mechanistically controls *ACE2* transcription; ATAC-seq only reflects bulk chromatin accessibility rather than direct transcription factor binding events. In addition, independent qPCR validation of *ACE2* expression could not be implemented in the present study. As an endangered protected species, blood sampling of giant pandas is highly restricted by conservation protocols, and all collected peripheral blood from the six experimental individuals was fully consumed for RNA-seq and ATAC-seq library preparation, leaving no residual specimen for orthogonal verification. We plan to continuously monitor captive geriatric giant pandas with stable hypertensive phenotypes and recruit additional age-matched normotensive controls in subsequent years, and we will perform qPCR validation of *ACE2* expression using newly acquired blood samples to verify the candidate molecular signature reported in this exploratory small cohort study.

The exploration and analysis of cell-type-specific marker genes have enabled us to uncover the complex changes in cell populations under pathological conditions such as hypertension. By annotating and classifying cells using specific marker genes, we identified 11 clusters, including the known monocytes, DCs, NK cells, various T cell subgroups, and multiple unknown cell clusters. This classification method relies on marker genes such as *IL7R*, *S100A8*, *FRY* and *KLRD1*, etc., to help identify specific immune cell types and highlight the potential function of each cell in immune responses and diseases [27]. However, this study lacks a species-matched giant panda immune cell reference transcriptome atlas for robust cell type classification. The putative dendritic cell populations (clusters 1, 11, 13, 28) were annotated based on non-canonical enriched genes FRY, NRGN and CADPS2, and this assignment carries moderate confidence and should be interpreted cautiously. Definitive DC identity validation will require future multi-species comparative immune profiling and immunophenotyping experiments. All poorly characterized unclassified cell clusters with low annotation confidence are listed in Supplementary Table S4, and we should avoid functional speculation for these ambiguous populations throughout the analysis. It is well acknowledged that transcriptional signals captured by bulk whole blood RNA-seq are strongly confounded by dynamic shifts in peripheral immune cell composition, which makes it difficult to distinguish whether gene expression changes originate from altered cell proportions or intrinsic transcriptional regulation within specific cell populations. To resolve this confounding factor, we leveraged our single-cell RNA-seq dataset to map the cell type distribution of *ACE2* and other hypertension-associated differentially expressed genes across all annotated immune clusters. Our research found that the differential genes related to blood pressure are concentrated in monocytes, T cells and unknown cells, revealing the key role of these cells in blood pressure regulation. For instance, genes such as *VAV3*, HDAC9 and *JAK2* play significant roles in immune responses and signal transduction, suggesting their contribution to the development of hypertension [28]. In addition, genes such as *STK39*, *KCNQ5* and *RAMP1* are associated with ion channels and signaling pathways, suggesting that the dysfunction of ion channels may be associated with an important mechanism of hypertension [29]. However, we did not detect specific expression of *ACE2* in monocytes or T cells at single-cell resolution; *ACE2* transcripts were barely detectable across the major circulating immune cell clusters profiled herein. This discrepancy between bulk and single-cell transcriptomic profiles implies that the overall reduction in *ACE2* abundance observed in bulk peripheral blood may not stem from intrinsic transcriptional suppression within mainstream monocytic and T lymphocyte populations. Instead, the bulk-level *ACE2* downregulation is likely driven by rare non-lymphoid blood cell populations, or indirectly mediated by systemic inflammatory remodeling of peripheral immune microenvironment under hypertensive conditions. The GO and KEGG analyses further supported the enrichment of these genes in the structural and functional participation of ribosomes, highlighting the significance of protein synthesis and its regulation in the pathology of hypertension. The enrichment of ribosome-related pathways may indicate metabolic adjustments in cells in response to hypertensive stress, suggesting that specific protein translation processes may be affected, thereby participating in the progression and regulation of the disease [30].

The accuracy of cell subtype classification in this study is constrained by the extreme scarcity of species-specific immune transcriptomic resources for giant pandas. No dedicated giant panda immune single-cell reference atlas has been established to enable high-confidence label transfer. Although we integrated conserved mammalian canonical immune markers and cluster-specific signature genes for cell assignment, many classic human and mouse immune marker homologs show weak or absent expression in giant panda peripheral blood cells, limiting their utility for annotation. Accordingly, multiple cell clusters could not be reliably assigned to known immune subtypes and were labeled as “unknown”. For the identified dendritic cell population, FRY, NRGN and CADPS2 were the most statistically specific signature genes detected in our dataset, rather than canonical DC markers commonly reported in model mammals. Future large-scale single-cell profiling of giant panda immune cells across more individuals will help build a species-specific reference atlas, resolve uncharacterized unknown clusters, and validate cell-type-specific marker genes unique to giant pandas.

We only described visual trends in cell type relative abundance across samples without formal compositional statistical analysis to quantify group differences. Moreover, the study cohort is limited to merely three giant pandas in each group, which results in low statistical power to distinguish hypertension-specific cell composition shifts from natural inter-individual variation. The visually observed divergent trends in cell abundance should only be regarded as preliminary observational clues rather than reliable disease-linked signatures. Expanded cohorts of age-matched hypertensive and normotensive giant pandas are needed in follow-up research to conduct rigorous compositional analysis and validate whether immune cell composition correlates with hypertensive phenotypes.

Our study included only six giant pandas, a small cohort that limits statistical power and the generalizability of our correlative molecular signatures. Constraints on sampling endangered giant pandas prevent recruitment of a larger sample set at this stage. Thus, all candidate hypertension-associated transcriptional and chromatin signatures discovered in this exploratory multi-omics work necessitate follow-up validation in enlarged independent giant panda cohorts before definitive biological interpretations can be drawn. In addition to the limited sample size, chronological age constitutes an important biological confounder between our hypertensive and normotensive cohorts. The three hypertensive giant pandas were born earlier than the normotensive individual. Notably, per the standard age grading criteria for captive giant pandas, all six experimental animals are classified as geriatric individuals, meaning no juvenile or middle-aged pandas were enrolled in this study, and all molecular profiling was performed exclusively on aged subjects. However, the age gap between groups still prevents us from completely separating hypertension-specific transcriptional and chromatin remodeling from progressive age-related molecular shifts in peripheral blood. In captive breeding systems, spontaneous sustained hypertension is almost exclusively diagnosed in geriatric giant pandas. Normotensive giant pandas of the exact same advanced age as our hypertensive cohort are extremely scarce and unavailable for sampling at present. We propose that future multi-omics studies recruiting perfectly age-balanced elderly hypertensive and normotensive giant pandas will be required to fully decouple progressive aging molecular changes from hypertension-specific alterations.

Our longitudinal RNA-seq profiling of hypertensive giant pandas following levamlodipine administration carries prominent interpretive limitations originating from the sampling schedule. We only collected a single baseline sample (h0) right before initial drug intake, without a prolonged series of matched no-drug baseline time points from identical individuals to establish rigorous within-animal self-control. Parallel untreated hypertensive cohorts and placebo groups were also unavailable due to giant panda conservation and veterinary clinical safety restrictions. This design deficit prevents us from fully disentangling levamlodipine-specific transcriptional responses from time-dependent background variation, including cumulative stress from repeated venipuncture, diurnal circadian transcriptional oscillation, consistent feeding routines and standardized animal handling procedures. Accordingly, all time-series gene expression patterns reported herein are only described as sequential molecular alterations observed across sampling time points after levamlodipine administration, rather than confirmed drug-specific pharmacodynamic transcriptional signatures. Future intervention studies on hypertensive giant pandas will integrate multiple matched pre-treatment baseline samples for each individual to strengthen the discrimination of drug-triggered molecular signals from non-specific temporal background fluctuations.

In this study, levamlodipine besylate tablets were orally administered to hypertensive giant pandas for routine clinical antihypertensive management, and serial peripheral blood RNA-seq was performed to characterize time-resolved transcriptomic alterations following drug exposure. Differential expression analysis identified elevated transcript levels of SLC39A8 and SLC16A11 from h0 to h6, corresponding to enrichment of metabolic biological processes. GO functional enrichment highlighted prominent enrichment of phosphate hydrolysis and oxygen transport activities, reflecting shifts in transcriptional programs linked to cellular metabolic and oxygen transport homeostasis across this early-to-middle sampling window. We also observed sustained reduced transcript abundance of RYR2 and KCNJ5 from mid to late time points, two genes functionally associated with calcium channel activity. This expression trend overlaps with suppressed transcriptional signatures of intracellular calcium storage and calcium signaling pathways reported in prior mammalian research [31]. Time-series functional enrichment further revealed dynamic shifts in activated biological programs across sampling time points: early-stage transcriptional upregulation centered on oxygen transport and synaptic assembly processes, while later time points featured enhanced transcription linked to protein synthesis and ribosomal function. These sequential molecular shifts reflect layered, time-ordered transcriptional remodeling in peripheral blood cells following levamlodipine administration, capturing a progressive cellular transcriptional adaptation process across the 24 h sampling window. Notably, this dataset lacks synchronized serial blood pressure measurements collected at each RNA-seq time point, so the detected time-dependent transcriptomic changes cannot be definitively linked to levamlodipine-induced blood pressure reduction or validated antihypertensive pharmacodynamic effects. All observed gene and pathway transcriptional trajectories only represent descriptive molecular profiles captured after drug administration, and do not serve as direct evidence of phenotypic cardiovascular regulation or hypotensive activity. Future matched multi-omics research integrating parallel dynamic blood pressure monitoring will be required to verify whether these temporal transcriptional signatures correlate with actual hemodynamic improvements in hypertensive giant pandas. Further mechanistic exploration of these time-resolved molecular patterns may help inform optimized clinical medication regimens and improve understanding of long-term levamlodipine molecular responses in captive giant pandas.

## 5. Conclusions

This study identified exploratory blood-based molecular signatures linked to hypertension in a small cohort of geriatric giant pandas. The *ACE2* gene, whose differential chromatin signal was detected in exon regions, exhibited reduced expression in hypertensive giant pandas and participates in modulation of the renin–angiotensin system. Differentially expressed genes enriched in ribosome pathways were predominantly distributed in monocytes, T cells and several uncharacterized immune cell subsets. We observed time-dependent transcriptional changes following levamlodipine besylate administration. Genes *SLC39A8* and *SLC16A11* were upregulated during the early drug response phase accompanied by prominent activation of metabolic pathways. By contrast, the late treatment stage was characterized by downregulated *RYR2* and *KCNJ5* expression, alongside suppressed ion channel and calcium signaling cascades. Collectively, our findings identify preliminary time-resolved peripheral blood transcriptomic alterations detected after levamlodipine besylate administration in hypertensive giant pandas, providing descriptive gene-level molecular profiles captured across sequential sampling windows. As an exploratory analysis based on peripheral blood transcriptomic and chromatin data from limited aged giant panda individuals, this work provides preliminary scientific reference for optimizing targeted antihypertensive strategies and improving long-term health management of captive giant pandas.

## Figures and Tables

**Table 1:** Information on giant pandas in this experiment.

Name	Stud#	Sex	Age/Group	Mean Systolic Blood Pressure (mmHg)	Mean Diastolic Blood Pressure (mmHg)	Number of Measurements	Clinical Signs and Relevant Medical History	Drug Feeding Concentration
Ximeng	399	Male	31/elderly	179.7 ± 6.51	157.3 ± 18.15	9	hypertensive	0.025 mg/kg
Shulan	407	Female	32/elderly	180.3 ± 0.58	140.7 ± 4.04	9	hypertensive	0.025 mg/kg
Gaogao	415	Male	35/elderly	180 ± 3.61	153.3 ± 10.5	9	hypertensive	0.025 mg/kg
Zhangka	505	Female	25/elderly	114.7 ± 4.16	108 ± 12.12	9	normotensive	0 mg/kg
Yangyang	579	Male	24/elderly	110.7 ± 10.79	108 ± 2.65	9	normotensive	0 mg/kg
Tiantian	458	Male	28/elderly	103.7 ± 3.06	98 ± 8.54	9	normotensive	0 mg/kg

## Data Availability

The datasets generated and/or analyzed during the current study are available in the Genome Sequence Archive (GSA), and the accession numbers were CRA043593 (ATAC-seq) and CRA043595 (scRNA-seq).

## Supplementary Materials

The following supporting information can be downloaded at: https://www.mdpi.com/article/10.3390/ani16142116/s1, Figure S1: Spearman correlation of read counts; Table S1: Quality control statistics for RNA-seq data; Table S2: Quality statistics of ATAC-seq data; Table S3: Cell quantity statistics of scRNA-seq data; Table S4: Summary of cell-type annotation evidence for all scRNA-seq clusters.
